# Plant root transcriptome profiling reveals a strain-dependent response during *Azospirillum*-rice cooperation

**DOI:** 10.3389/fpls.2014.00607

**Published:** 2014-11-06

**Authors:** Benoît Drogue, Hervé Sanguin, Amel Chamam, Michael Mozar, Christel Llauro, Olivier Panaud, Claire Prigent-Combaret, Nathalie Picault, Florence Wisniewski-Dyé

**Affiliations:** ^1^Ecologie Microbienne, UMR CNRS 5557/USC INRA 1364, Université Lyon 1Villeurbanne, France; ^2^Laboratoire de Génome et Développement des Plantes, UMR 5096 CNRS/IRD/Université de Perpignan Via DomitiaPerpignan, France

**Keywords:** *Azospirillum*, hormone signaling, plant defense, plant growth-promoting rhizobacteria, rice, transcriptome

## Abstract

Cooperation involving Plant Growth-Promoting Rhizobacteria results in improvements of plant growth and health. While pathogenic and symbiotic interactions are known to induce transcriptional changes for genes related to plant defense and development, little is known about the impact of phytostimulating rhizobacteria on plant gene expression. This study aims at identifying genes significantly regulated in rice roots upon *Azospirillum* inoculation, considering possible favored interaction between a strain and its original host cultivar. Genome-wide analyzes of *Oryza sativa japonica* cultivars Cigalon and Nipponbare were performed, by using microarrays, seven days post-inoculation with *Azospirillum lipoferum* 4B (isolated from Cigalon) or *Azospirillum sp*. B510 (isolated from Nipponbare) and compared to the respective non-inoculated condition. A total of 7384 genes were significantly regulated, which represent about 16% of total rice genes. A set of 34 genes is regulated by both *Azospirillum* strains in both cultivars, including a gene orthologous to PR10 of *Brachypodium*, and these could represent plant markers of *Azospirillum-rice* interactions. The results highlight a strain-dependent response of rice, with 83% of the differentially expressed genes being classified as combination-specific. Whatever the combination, most of the differentially expressed genes are involved in primary metabolism, transport, regulation of transcription and protein fate. When considering genes involved in response to stress and plant defense, it appears that strain B510, a strain displaying endophytic properties, leads to the repression of a wider set of genes than strain 4B. Individual genotypic variations could be the most important driving force of rice roots gene expression upon *Azospirillum* inoculation. Strain-dependent transcriptional changes observed for genes related to auxin and ethylene signaling highlight the complexity of hormone signaling networks in the *Azospirillum*-rice cooperation.

## Introduction

Rhizodeposition supports growth of a wide range of microorganisms able to establish intimate interactions with plant roots. In the case of parasitism, nutritional requirements of the microbe partner are supported at the expense of plant development and reproduction (O'Brien et al., 2011; Schumacher and Tudzynski, 2012). In the case of mutualism, the interaction leads to a nutritional exchange so that costs and benefits are reciprocally shared by both microbial and plant partners (Smith and Read, 2008). Whether engaged in a parasitic or mutualistic interaction, the microbial partner is perceived as an intruder and the success of the adaptation strategy partly depends on the microbe's ability to bypass defense mechanisms and invade plant tissues (Soto et al., 2009). Then, plant immune response involves gene expression changes that mediate trade-off between defense and development to ensure plant survival through an efficient allocation of resources (Buscaill and Rivas, 2014). Cooperation involving Plant Growth-Promoting Rhizobacteria (PGPR) results in improvements of plant growth and health; however, the invasion of root tissues is not a critical step in successful interaction as several efficient strains are described as root-surface colonizers (Lugtenberg and Kamilova, 2009; Chamam et al., 2013). If mechanisms directly implicated in plant growth-promotion have been extensively studied, most of these works have assessed the impact of PGPR on plant morphological traits and little is known about changes induced at the molecular level (Bashan and de-Bashan, 2010; Galland et al., 2012; van de Mortel et al., 2012; Wisniewski-Dyé et al., 2013).

For more than 50 years, PGPR of a wide range of genera including *Acetobacter, Azospirillum, Bacillus, Burkholderia, Herbaspirillum, Phyllobacterium* or *Pseudomonas* have been known for stimulating the growth of numerous host plants (Desbrosses et al., 2009; Lugtenberg and Kamilova, 2009; Richardson et al., 2009; Kumar et al., 2011; Saharan and Nehra, 2011). In particular, the genus *Azospirillum* constitutes an important phytostimulator and an increasing number of field trials are undertaken, principally in India and Latin America where several *Azospirillum* inoculants are commercialized (Steenhoudt and Vanderleyden, 2000; Bashan et al., 2004; Fuentes-Ramirez and Caballero-Mellado, 2012). In most cases, successful inoculation results in root and shoot morphological changes, plant nutrition improvements, and yield enhancements (Richardson et al., 2009; Vacheron et al., 2013; Wisniewski-Dyé et al., 2013). If the phytostimulating effect of *Azospirillum* was originally attributed to its ability to fix atmospheric nitrogen, it is now admitted that the modulation of the phytohormonal balance is the most important mechanism resulting in the modification of root architecture and higher nutrient uptake by the plant (Steenhoudt and Vanderleyden, 2000; Somers et al., 2004; Prigent-Combaret et al., 2008). Besides morphological changes, *Azospirillum* also increases root exudation and modifies the chemical structure of root cell wall (Heulin et al., 1987; El Zemrany et al., 2007). In addition, *Azospirillum* was found capable of increasing the resistance of the host plant against pathogen through mechanisms independent of salicylic acid signaling (Yasuda et al., 2009). Investigation on maize secondary metabolism revealed that major qualitative and quantitative modifications occur following *Azospirillum* inoculation (Walker et al., 2011). Moreover, these modifications depend on bacterial strain/maize cultivar combinations suggesting that a genotype specific perception of *Azospirillum* occurs during the cooperation with maize. These observations were recently strengthened by a study made on two rice cultivars, Cigalon and Nipponbare, after the inoculation of two *Azospirillum* strains isolated from each cultivar (Chamam et al., 2013): *Azospirillum lipoferum* 4B isolated from Cigalon roots (Thomas-Bauzon et al., 1982) and *Azospirillum* sp. B510 isolated from Nipponbare (Elbeltagy et al., 2001). Profiling of secondary metabolites and morphological measurements evidenced that the impact of *Azospirillum* differs according to strain/cultivar combinations and that a specific interaction leading to a stronger phytostimulation occurs between a strain and its original host cultivar. In addition, the endophyte strain B510 was shown to trigger a systemic response, as metabolic changes were observed in both roots and shoots. However, whether or not perception of *Azospirillum* involved plant immune response remains an unanswered question and regulatory mechanisms underlying host-specific metabolic changes have to be unraveled.

In this context, our study aims at characterizing genetic determinants regulated in rice roots at an early stage of the interaction with *Azospirillum*, considering possible favored interaction between a strain and its original host cultivar. Thus, genome-wide analyzes of root gene expression of *Oryza sativa japonica* cultivars Cigalon and Nipponbare were performed 7 days post-inoculation with *A. lipoferum* 4B or *Azospirillum* sp. B510 and compared to the respective non-inoculated condition. A focus was made on genes potentially involved in plant defense and hormone signaling.

## Materials and methods

### Biological material

In this study, two rice (*Oryza sativa* L.) cultivars belonging to the *japonica* group, cv. Cigalon (Center Français du Riz, France) and cv. Nipponbare (J. B. Morel, BGPI, Montpellier, France) were inoculated with two diazotrophic strains of the genus *Azospirillum*: *A. lipoferum* 4B initially isolated from rice roots of the cv. Cigalon in France (Thomas-Bauzon et al., 1982) and *Azospirillum* sp. B510 initially isolated from surface sterilized rice stems of the cv. Nipponbare (Elbeltagy et al., 2001).

### RNA samples and cDNA synthesis

Six independent experiments were performed per condition (three for microarray hybridization and three for qRT-PCR validation). Seed sterilization, plant inoculation and plant growth were performed as previously described (Chamam et al., 2013; Drogue et al., 2014). Rice seeds were surface sterilized by washing for 40 min in a sodium hypochlorite solution, rinsed 5 times in demineralized sterile water, and then chlorine traces were removed by washing 3 times in sterile-filtered 2% (w/v) sodium thiosulfate before rinsing 5 times in demineralized sterile water. Surface sterilized seeds were germinated on sterile plant agar (8 g·L^−1^) (Sigma Chemical Co, Saint Louis, USA) for 2 days in the dark at 28°C. Bacterial cells in late-exponential phase were mixed with 50 mL of plant agar (8 g·L^−1^) (to a final concentration of 2.10^7^ cells·mL^−1^) and introduced into 120 × 120 × 17 mm square plates. For both rice cultivars, five disinfected germinated seeds were laid onto the plates and plates were incubated vertically, for 7 days in a growth chamber (MLR350, SANYO, UK) with a photoperiod of 16 h at 28°C (light 150 μE m^−2^ s^−1^), and 8 h at 22°C in the dark. For each experiment, 30 plant root systems were pooled and frozen using liquid nitrogen. Root cell lysis was performed by grinding root systems with a mortar and pestle under liquid nitrogen. Total RNA was isolated using the TRIzol method (Invitrogen, Carlsbad, CA, USA). RNA samples were purified using RNeasy plant mini kit (Qiagen, Courtaboeuf, France) according to the manufacturer's protocol. RNA integrity was assessed using Agilent RNA 6000 Pico Kit (Agilent Technologies, Waldbronn, Germany) and the Agilent 2100 Bioanalyzer (Agilent Technologies) device.

In order to increase mRNA representation in RNA samples, total RNA were digested with mRNA ONLYTM Procaryotic mRNA isolation kit (Epicenter Biotechnologies, Madison, WI, USA) according to the provided protocol.

The microarray cDNA (three independent samples per condition) was synthesized with the Superscript® Double-Stranded cDNA Synthesis Kit (Invitrogen), using a mix (1:1) of random primers (Promega Corporation, Madison, WI, USA) and Oligo-dT (15) primers (Promega).

### Microarray hybridization and data analysis

We designed an oligo microarray, which was produced by NimbleGenTM (Madison, WI, USA) derived of one which was described previously (Picault et al., 2009). This microarray is composed of about 385,000 60 mer probes selected for their GC content, Tm, and number of cycles needed to synthesize the oligo. This chip contains 90,000 probes representing 45,000 genes (two probes per gene) of rice *Oryza sativa* ssp. *japonica*, based on the TIGR rice genome annotation version 3.1 genes (Yuan et al., 2005) and 201,691 oligomers corresponding to previously described copies of LTR retrotranposons available on the retrOryza database (www.retroryza.fr; Chaparro et al., 2007). Probes represent 1000 bp of the LTR-retrotransposon flanking regions at the 3′ and 5′ side. The oligonucleotides have been designed at the 3′ end of the genes to detect the readings of reverse transcriptase. On the other hand, the LTR-retrotransposons are represented throughout their length at the rate of a probe every 500 bp. The analysis was performed using the classification proposed by El Baidouri and Panaud (2013) that consists in 369 families and 3623 loci harboring complete elements. Among the differentially expressed oligomers, only those displaying 100% identity and which are unique in the genome were analyzed.

When it was possible, probes have been designed to be unique in the genome (i.e., locus specific) to overcome the problems of oligonucleotides redundancy on the chip. When there were three mismatches during hybridization between a cDNA and an oligonucleotide, hybridization was considered stable enough to withstand the conditions of washing after the chip hybridization. The oligonucleotides are therefore regarded as locus specific when they are not matching elsewhere, but having at most three mismatches, which represents 5% of all oligonucleotides.

For each condition, three independent cDNA samples were labeled and hybridized by Roche Nimblegen according to their standard protocol. Data analysis was performed using Bioconductor microarray packages for R software (http://www.bioconductor.org/). The robust multi-array average (RMA) method associated with quantile normalization was applied (Bolstad et al., 2003; Irizarry et al., 2003). Analysis of variance with a false discovery rate adjustment method was realized (Benjamini and Hochberg, 1995). The results of different treatment comparison were obtained in Log2-fold change. The oligonucleotides selected were those which present a two fold increase or decrease in expression, i.e., a log-fold change smaller or equal to −1 for down-regulation, and greater or equal to 1 for up-regulation. Oligonucleotides displaying *P* ≤ 0.05 for the statistical test were selected. For each cultivar, the respective uninoculated condition was used as control. All oligonucleotides differentially expressed were remapped to Os-Nipponbare-Reference-IRGSP version 1.0 (Rice Annotation Project et al., 2008) using BLAST.

### RT-qPCR

For each condition, three independent RNA samples were used to validate gene expression level by performing reverse transcription quantitative real-time PCR (RT-qPCR). Validation was made on a group of 14 representative genes (Table 1) using LightCycler® 480 SYBR Green I Master kit (Roche Diagnostics GmbH, Mannheim, Germany) on a LightCycler® 480 Real-Time PCR System (Roche). The actin gene (Os03g0718100) showing an invariant expression was used as reference to normalize RT-qPCR values. After DNase I treatment, total RNA (800 ng) was used for cDNA synthesis using GoScriptTM Reverse transcription system (Promega) with oligo-dT (15) primer in accordance with the manufacturer's protocol.

**Table 1:** **Validation of microarray data**.

**Gene**		**FC Cig_4B^a^**		**FC Cig_B510**		**FC Nip_4B**		**FC Nip_B510^b^**	
		**Log_2_ (qPCR)**	**Log_2_ (array)**	**Log_2_ (qPCR)**	**Log_2_ (array)**	**Log_2_ (qPCR)**	**Log_2_ (array)**	**Log_2_ (qPCR)**	**Log_2_ (array)**
**Differentially expressed in all conditions**									
Os09g0358000	Tyrosine kinase domain	2.27	1.77	2.53	2.46	1.87	3.01	2.06	1.32
**Differentially expressed in Cig_4B and Nip_4B only**									
Os09g0417800	WRKY transcription factor 62	1.49	1.75	0.66	_	1.00	1.88	−0.01	_
Os11g0686900	Similar to NB-ARC domain	0.83	1.16	−0.23	_	1.74	2.40	nd	_
**Differentially expressed in Cig_B510 and Nip_B510 only**									
Os06g0115600	Common symbiosis signaling (SYM) pathway	−0.09	_	−2.00	−1.84	0.01	_	−1.18	−1.47
Os05g0583000	Similar to WRKY8	0.32	_	2.92	1.71	0.16	_	2.24	1.39
Os12g0139400	A-type response regulator, cytokinin signaling	−0.15	_	−2.84	−1.78	−0.09	_	−2.94	−1.10
Os11g0143300	A-type response regulator, cytokinin signaling	0.15	_	−1.09	−1.95	0.04	_	−2.25	−1.62
Os02g0805100	Similar to auxin-responsive protein IAA12	−0.01	_	−1.15	−1.34	−0.23	_	−2.47	−1.16
**Differentially expressed in Cig_4B and Cig_B510 only**									
Os05g0196600	Similar to ACC synthase	0.38	1.33	0.95	1.63	−0.15	_	0.16	_
**Differentially expressed in Cig_4B only**									
Os08g0136100	Homeobox-leucine zipper domain	0.87	1.11	0.25	_	0.28	_	−0.14	_
**Significantly regulated in Cig_B510 only**									
Os06g0179200	Similar to Nodulin-like protein	0.16	_	−1.60	−1.78	0.18	_	0.12	_
Os08g0499300	WRKY transcription factor 30	0.10	_	0.52	1.34	0.12	_	−0.06	_
**Significantly regulated in Nip_B510 only**									
Os01g0904700	B-type response regulator, cytokinin signaling	−0.12	_	−0.07	_	−0.04	_	−1.09	−1.21
Os05g0515400	Similar to auxin response factor 14	−0.09	_	0.10	_	−0.09	_	−1.15	−1.42
**Reference gene**									
Os03g0718100	Actin								

a *Dashes replace non-significant fold change values obtained from microarray data (|Log_2_(FC)| ≤ 1 and P_adj_ > 0.05)*.

b *nd, not determined*.

DNA contamination was checked with reactions that lacked reverse transcriptase as negative controls. Specific primers were designed using Light Cycler Probe design Software 2.0 (Roche) with the following criteria: product size ranges 100–400 pb, primer size comprised between 17 and 22 bases, optimal primer Tm 60°C (Additional file 1: Table S1). Real time PCR conditions were: a denaturation stage of 10 min at 95°C; an amplification stage of 45 cycles of 15 s at 94°C, 10 s at 60°C and 20 s at 72°C; and a melting curve stage of 5 s at 95°C and 1 min at 65°C increased to 97°C with a ramp rate of 0.11°C s^−1^. All reactions were performed in three technical replicates and carried out in LightCycler 480 Multiwell plate 96 (Roche) with adhesive sealings foils (Roche) in a final volume of 10 μl containing 1 μl of each primer (5 μM), 5 μl of master mix and 3 μl of cDNA diluted 50 times. For each cultivar, the respective uninoculated condition was used as the calibrator condition and relative gene expression was calculated using the 2^−ΔΔ*Ct*^ method (Livak and Schmittgen, 2001).

Data were statistically validated by a correlation test using the Pearson's method.

### Availability of microarray data

Microarray data are available on GEO database through the following accession number GSE59137.

## Results

Transcriptomic profiles were obtained using microarrays and the four following combinations were analyzed (three independent replicates per combination): Cigalon/*A. lipoferum* 4B (Cig_4B) and Cigalon/*Azospirillum* sp. B510 (Cig_B510) compared to non-inoculated Cigalon; Nipponbare/*A. lipoferum* 4B (Nip_4B) and Nipponbare/*Azospirillum* sp. B510 (Nip_B510) compared to non-inoculated Nipponbare. Genes significantly regulated were selected using a *P*_adjusted_-value (*P*_adj_) threshold of 0.05 and a fold change cutoff of 2 (|Log_2_(FC)| ≥ 1). According to the cultivar of which each strain was originally isolated, Cig_4B and Nip_B510 combinations constitute interactions between a strain and its original host cultivar, which will be hereafter designed as host combinations, while Cig_B510 and Nip_4B combinations constitute interactions with non-host cultivars, which will be hereafter designed as non-host combinations.

### General features of rice-root transcriptome profiling in response to *Azospirillum* inoculation

Microarray design was based on the genome sequence of cultivar Nipponbare. To ensure that it could be used to hybridize cDNA obtained from cultivar Cigalon, the genetic proximity between both cultivars was analyzed by sequencing eight genes (including the gene encoding actin) after PCR amplification from Cigalon DNA (at least 500 pb per gene) (Additional file 2: Table S2). For all the sequenced genes, an identity of 100% was observed with the corresponding genes of Nipponbare. In addition, when comparing the non-inoculated Cigalon transcriptome profile to the non-inoculated Nipponbare profile, only 193 genes are differentially transcribed between the two cultivars (87 up-regulated and 106 down-regulated). This represents only 0.43% of the targeted genes (i.e., 45,000 genes) and may be explained by physiological differences. Thus, the microarray was considered suitable for analysis and comparison of both Nipponbare and Cigalon transcriptomes.

**Figure 1:**
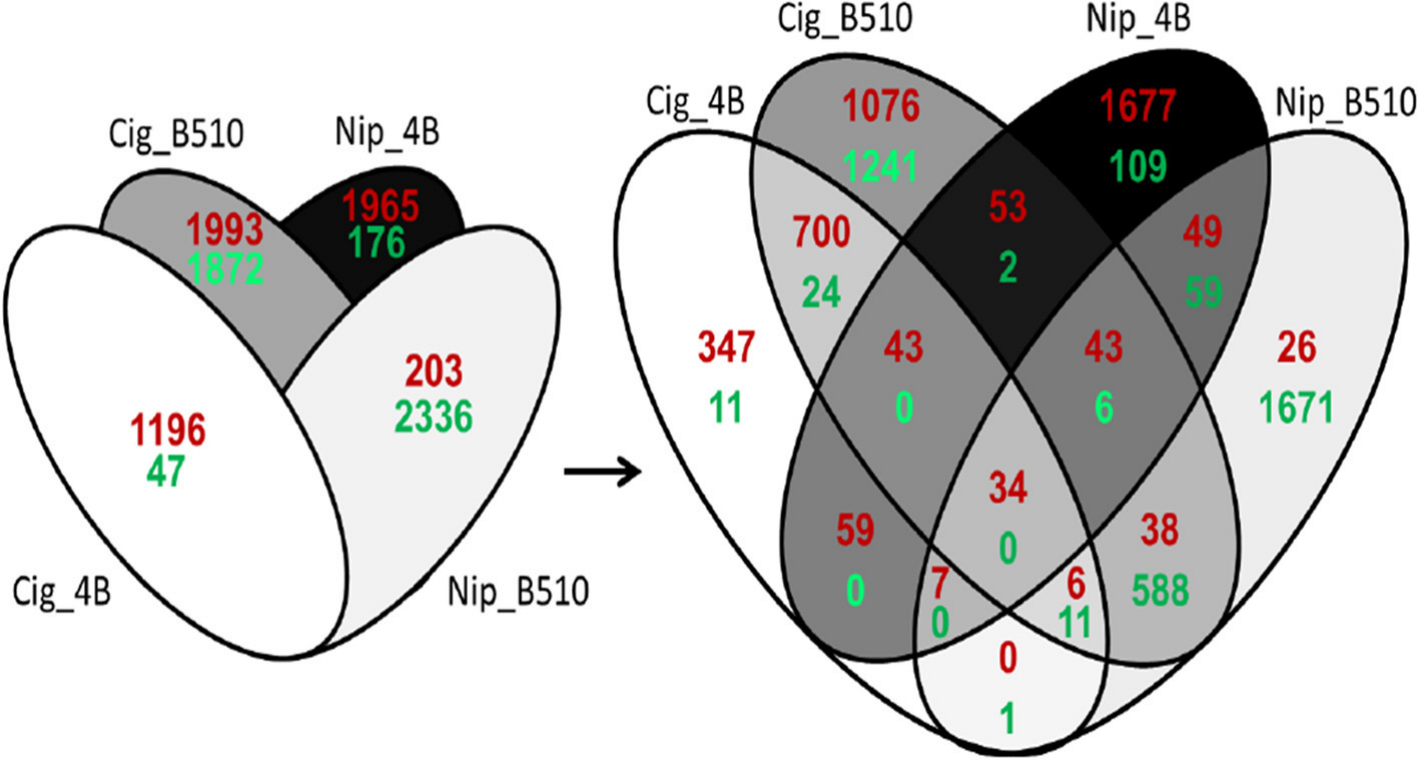
**Venn diagram of genes differentially expressed in rice roots (|Log_2_(FC)| ≥ 1 and *P*_adj_ ≤ 0.05)**. *Oryza sativa* L. *japonica* cultivar Cigalon and cultivar Nipponbare were inoculated with *Azospirillum* strains 4B and B510. For each cultivar, the respective non-inoculated condition was used as reference to evidence genes up-regulated (red) and down-regulated (green) after *Azospirillum* inoculation (|Log_2_(FC)| ≥ 1 and *P*_adj_ < 0.05). Cig_4B and Nip_B510 combinations constitute the interaction between a strain and its original host cultivar (host combinations). Cig_B510 and Nip_4B combinations constitute interactions with non-host cultivars (non-host combinations).

When considering the four combinations, a total of 7384 genes are differentially expressed in rice roots, which represent about 16% of the entire set of rice genes. Each strain/cultivar combination displays specific expression profiles highlighting a strain-specific response of the host plant. The most important changes are observed when strain B510, isolated from Nipponbare, is inoculated on Cigalon roots (Cig_B510), with 3865 regulated genes, equally induced and repressed (1993 up-regulated; 1872 down-regulated) (Figure 1). Conversely, the inoculation of strain 4B on its original cultivar Cigalon (Cig_4B) is accompanied by the differential expression of only 1243 genes, mostly induced (1196 up-regulated; 47 down-regulated). When considering Nipponbare roots, the number of regulated genes is similar for Nip_4B and Nip_B510 combinations with respectively, 2141 and 2539 regulated genes. However, these genes are mostly induced in Nip_4B (1965 up-regulated; 176 down-regulated) while they are repressed in Nip_B510 combination (203 up-regulated; 2336 down-regulated).

A Venn diagram analysis conducted on expression profiles obtained for the four combinations, unveils 15 sets of genes (Figure 1). Four sets, named combination-specific genes (genes induced or repressed only in one combination), represent 83% of all differentially expressed genes, with 358 genes for Cig_4B (347 up-regulated, 11 down-regulated), 2317 genes for Cig_B510 (1076 up-regulated, 1241 down-regulated), 1786 genes for Nip_4B (1677 up-regulated, 109 down-regulated) and 1697 genes for Nip_B510 (26 up-regulated, 1671 down-regulated) (Additional file 3: Table S3). Two other sets regroup genes that are common only to Cig_4B and Cig_B510 (700 up-regulated, 24 down-regulated) or only to Nip_4B and Nip_B510 (49 up-regulated, 59 down-regulated), representing *Azospirillum*-regulated cultivar-specific genes (Additional File 4: Table S4). Inversely, two sets comprise strain-specific genes that display the same regulation only in both Cig_4B and Nip_4B (59 up-regulated, zero down-regulated) or only in both Cig_B510 and Nip_B510 (38 up-regulated, 588 down-regulated) (Additional File 5: Table S5). Two additional sets include genes displaying the same regulation in both host combinations, or in both non-host combinations (Additional File 6: Table S6). One set contains 34 up-regulated genes common to the four combinations and no down-regulated genes. The four remaining sets comprise genes that are common to three of the four combinations and will no longer be discussed in the current analysis. Microarray data were confirmed by analyzing expression levels of 14 representative genes using reverse transcription quantitative polymerase chain reaction (RT-qPCR) (Table 1). This includes seven up-regulated genes and seven down-regulated genes belonging to seven of the 15 categories described in Figure 1. These results show that the array data are in accordance with the RT-qPCR data (*R*^2^ = 0.83; *P*_value_ = 2.7.10^−10^).

The transcriptome survey was completed with an analysis of the LTR-retrotransposons. Indeed, LTR-retrotransposons, a particular type of transposable elements, represent 25% of the total genomic sequence of rice (Rice Annotation Project et al., 2008). The transcriptional activation of LTR-retrotransposons can lead to the activation of a flanking gene, either through the action of the enhancer regions of the element or by co-transcription (Michaud et al., 1994); conversely, LTR-retrotransposons can also act as suppressors of gene expression when they are inserted in antisense in the 3′ region of a gene. Interestingly, *Azospirillum* inoculation leads to a differential expression of a large number of LTR-retrotransposon families for both rice cultivars. To avoid bias related to cultivar polymorphism, the complete analysis was performed only for the cultivar Nipponbare. For this latter, differential expression is observed for 115 and 148 LTR-retrotransposon families for strains B510 and 4B, respectively. In addition, strain 4B modifies the transcription of 21.4% of all LTR-retrotransposons, among which 51% are up-regulated and 49% down-regulated. For strain B510, 17.8% of all LTR-retrotransposons are differentially expressed (37% up-regulated and 62% down-regulated). Among the differentially expressed elements, 22% are common to Nip_4B and Nip_B510 combinations. In addition, we explored if transposable elements can significantly alter the expression of adjacent genes. In this analysis, two and six LTR-retrotransposons are located in the 3 kb upstream regions of regulated genes while four and five ones are located in the 3 kb downstream regions, for Nip_B510 and Nip_4B, respectively (Additional File 7: Table S7). However, no co-transcription events between gene and LTR-retrotransposon was evidenced by using RT-PCR method (data not shown), suggesting that none of these elements have a direct impact on the expression of adjacent genes.

### Impact of strain lifestyle on rice roots gene expression

**Figure 2:**
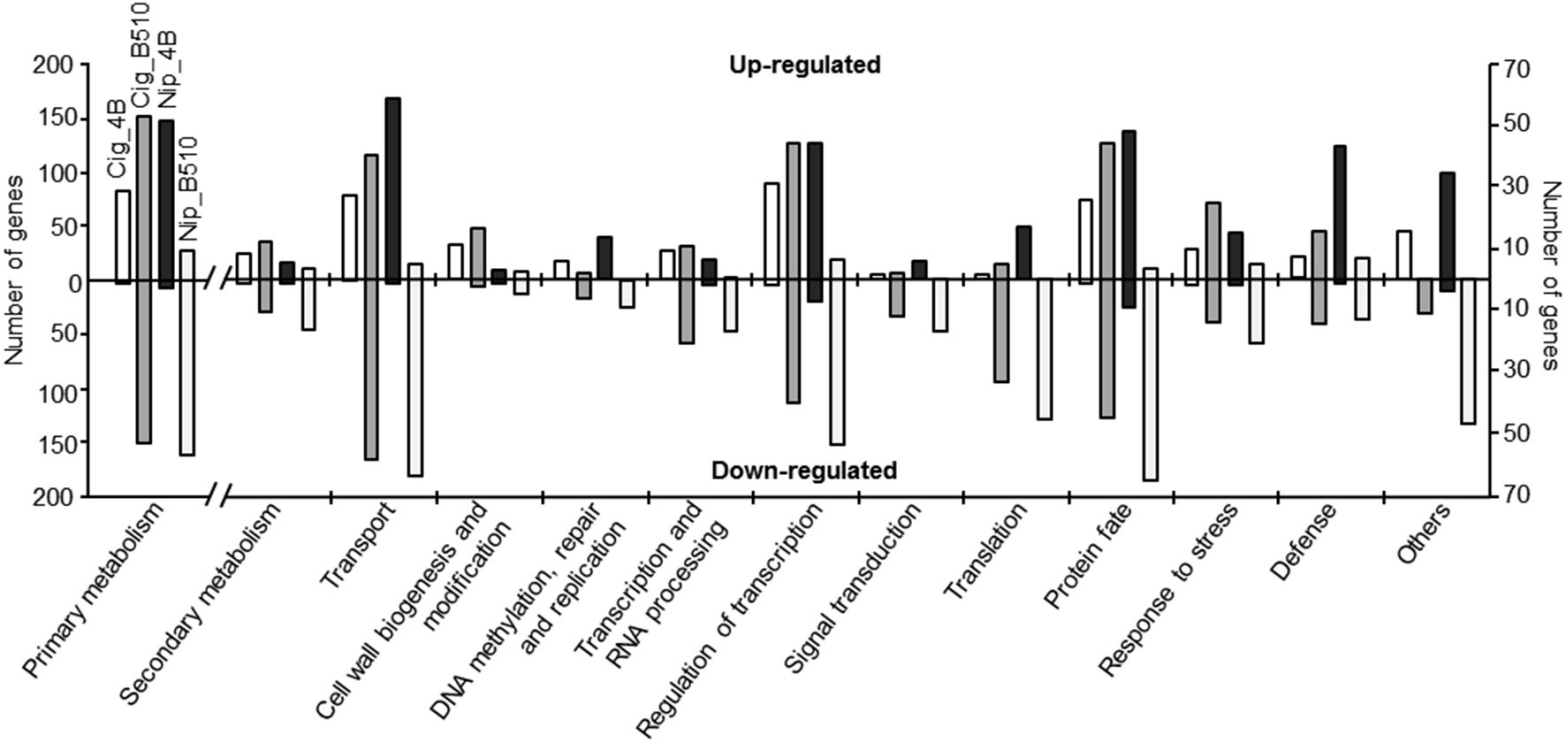
**Functional classification of the differentially expressed genes in *Oryza sativa* L. *japonica* in response to *Azospirillum* inoculation**. Numbers of genes differentially expressed are shown per functional categories for each *Azospirillum*-rice combination: Cig_4B (white), Cig_B510 (dark gray), Nip_4B (black), and Nip_B510 (light gray). Genes were classified according to Biological Process assignation taken from the Rap-DB database. Genes with no Biological Process assignation represent about 80% of differentially expressed genes for each condition. Category “Others” includes principally genes implicated in photosynthesis, exocytosis, sexual reproduction, cytoskeleton organization, cell cycle, and cell death.

**Figure 3:**
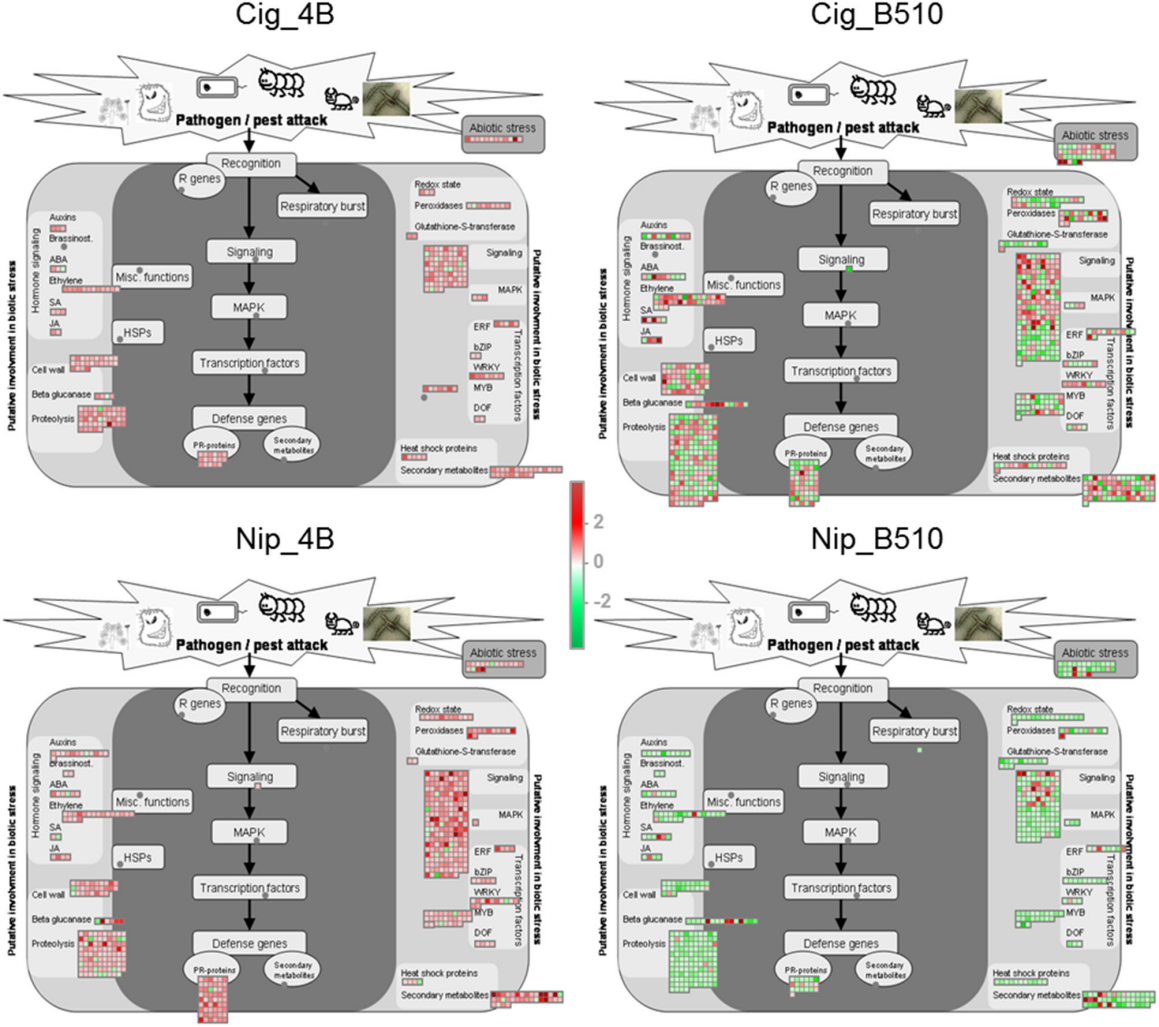
**Mapman software visualization of genes related to biological stress**. Genes differentially expressed (|Log_2_(FC)| ≥ 1 and *P*_adj_ ≤ 0.05) after *Azospirillum* inoculation on rice roots are represented according to their respective Log_2_ rescaled fold change. Red color represents up-regulated genes and green color represents down-regulated genes with the most intense color on the scale representing a Log_2_(FC) = 4.8. The dark gray rectangle includes genes directly involved in biotic stress responses and the light gray rectangle includes genes potentially involved in biotic stress response. ABA, abscisic acid; bZIP, basic region leucine zipper; DOF, DNA-binding with one finger; ERF, ethylene responsive factor; HSP, heat shock protein; JA, jasmonic acid; MAPK, mitogen activated protein kinase; MYB, myeloblast; PR, pathogenesis-related; R, resistance; SA, salicylic acid.

As mentioned above, most of the differentially expressed genes are induced in combinations involving strain 4B while they are mostly repressed for combinations involving strain B510 (Figure 1). In addition, when considering functional classification available for only 20% of all differentially expressed genes, several differences are evidenced at the strain level (Figure 2). Whatever the combination, the most important numbers of differentially expressed genes are observed for (i) primary metabolism, (ii) transport, (iii) regulation of transcription, and (iv) protein fate, four categories in which genes seem quasi exclusively repressed in Nip_B510 while they are quasi exclusively induced in Nip_4B. Similarly, when considering genes involved in response to stress and plant defense, two substantial categories in plant-microbe interactions, it appears that strain B510 leads to the repression of a more important number of genes than strain 4B. These results are of particular interest as they may be related to lifestyle differences between *Azospirillum* strains; indeed, strain 4B was shown to colonize only rice-root surface while strain B510 is able to colonize the outer layers of rice-root tissues (Elbeltagy et al., 2001; Chamam et al., 2013). To consider the impact of strain lifestyle on plant defense response, the analysis was focused on genes potentially involved in biotic stress. Identification of these genes was improved using Mapman annotation software as recently described for the *Azospirillum*-*Arabidopsis* interaction (Spaepen et al., 2014). This additional analysis, illustrated in Figure 3, confirms observations made above. Then, strain 4B leads quasi exclusively to the induction of genes related to biotic stress in Nipponbare and to a lower extent in Cigalon. On the contrary, genes involved in biotic stress response are quasi exclusively repressed in the Nip_B510 host combination, while the number of up-regulated genes and down-regulated genes is similar in the Cig_B510 non-host combination. In addition, Mapman visualization analysis highlights relevant categories of genes being differentially expressed such as genes encoding peroxidases, transcription factors of the MYB, WRKY, and ERF families as well as Pathogenesis-Related (PR) genes.

Another way to analyze the impact of strain lifestyle on rice roots gene expression was to consider the 4B_specific genes (59) and B510-specific genes (626) highlighted in the Venn diagram analysis (Figure 1; Additional File 5: Table S5). Among the 59 genes specifically induced by the surface-colonizing strain 4B, we identified 14 genes that could be involved in biotic stress response among which two genes encoding peroxidases, two WRKY transcription factors and one PR gene. While all the 4B-specific genes are up-regulated, 38 of the B510-specific genes are up-regulated and 588 are down-regulated including at least 14 and 102 genes associated to biotic stress response, respectively. The endophyte strain B510 triggers the induction of four genes potentially involved in hormone signaling, as well as genes encoding a peroxidase, a WRKY transcription factor and a PR protein. Moreover, a larger number of genes are down-regulated, including five PR genes, three genes encoding peroxidases but no WRKY transcription factor. Finally, the repression of a putative indole synthase (Os03g0797000), three putative auxin responsive proteins (Os02g0805100, Os08g0335600, Os11g0523800), two response regulators involved in cytokinin signaling (Os11g0143300, Os12g0139400), an isochorismate synthase (Os09g0361500) required for salicylic acid synthesis, as well as four genes potentially involved in ethylene signaling (Os03g0439500, Os04g0667400, Os09g0115500, 0s09g0309700) suggest that a B510-specific hormone signaling occurs in rice roots.

### Plant markers of *Azospirillum*-rice cooperation and similarities with pathogen infection

As revealed in the Venn diagram analysis (Figure 1), a total of 34 genes (12 genes of unknown function) are induced in the four combinations and could be defined as plant markers of *Azospirillum*-rice interactions (Table 2). Interestingly, a PR gene orthologous to PR10 of *Brachypodium* is induced in the four conditions (Os12g0555200). In addition, two genes encoding Cys-Rich domain containing protein (Os02g0579800, Os02g0580000) and an orthologous gene to AT1G59950 of *Arabidopsis thaliana* (Os03g0237100), which are potentially implicated in stress response, as well as a terpene synthase involved in gibberellin synthesis (Os04g0178300), and a gene encoding a putative precursor of phytoalexin (Os11g0474800, encoding a stemar-13-ene synthase) are induced in the four combinations. Finally, the set of potential plant markers of *Azospirillum*-rice cooperation also includes a transcription factor (Os01g0952800) and four genes potentially involved in signal transduction (Os07g0537900, Os08g0501500, Os08g0203400, Os09g0358000, this latter being validated by RT-qPCR, Table 1).

**Table 2:** **List of 34 genes induced in the four combinations**.

**Gene ID**	**Log_2_(FC)**				**Gene description**	**MSU Gene ID**	***M. oryzae*^a^**
	**Cig_4B**	**Nip_4B**	**Cig_B510**	**Nip_B510**			
Os01g0191200	1.34	3.28	2.18	3.09	Putative HAD phosphatase	LOC_Os01g09540	
Os01g0495701	1.14	2.60	2.02	2.12	Conserved hypothetical protein	None	
Os01g0608101	1.33	2.73	2.42	2.39	Conserved hypothetical protein	None	
Os01g0647200	1.07	2.97	1.91	1.71	Conserved hypothetical protein	LOC_Os01g45914	
Os01g0952800	1.70	3.32	2.75	2.65	Transcription factor	LOC_Os01g72370	
Os02g0569400	1.54	2.42	2.23	1.78	Cytochrome P450 family protein	LOC_Os02g36070	
Os02g0579800	1.49	3.08	1.75	1.91	Cys-rich domain containing protein	LOC_Os02g36940	+
Os02g0580000	1.51	2.80	1.78	1.32	Cys-rich domain containing protein	LOC_Os02g36950	
Os02g0582900	2.20	4.95	3.77	4.98	Conserved hypothetical protein	LOC_Os02g37190	+
Os02g0594232	1.18	2.28	2.09	1.8	Conserved hypothetical protein	None	
Os02g0791300	1.41	3.00	2.91	2.58	Conserved hypothetical protein	LOC_Os02g54870	
Os03g0129800	1.01	1.79	1.01	1.58	Uncharacterized protein	LOC_Os03g03730	+
Os03g0237100	1.42	3.43	2.40	2.94	Putative NADPH-dependent codeinone reductase	LOC_Os03g13390	
Os03g0307300	1.34	3.33	2.29	2.54	Nicotianamine synthase 1	LOC_Os03g19427	
Os04g0178300	1.25	3.22	2.39	2.91	Putative syn-copalyl diphosphate synthase	LOC_Os04g09900	
Os04g0178400	1.92	3.56	2.60	2.82	Putative cytochrome P450	LOC_Os04g09920	+
Os06g0293500	2.40	3.26	2.62	1.73	Conserved hypothetical protein	LOC_Os06g18960	+
Os06g0486800	1.10	2.31	1.84	1.42	Putative mitochondrial formate dehydrogenase	LOC_Os06g29180	
Os06g0718400	1.39	1.68	1.66	1.53	Plastocyanin-like domain containing protein	LOC_Os06g50420	
Os07g0190000	1.69	3.02	2.77	2.85	Putative 1-deoxy-D-xylulose 5-phosphate synthase	LOC_Os07g09190	+
Os07g0258400	1.61	2.25	2.60	1.70	Putative metal transporter Nramp6	LOC_Os07g15460	
Os07g0416900	2.76	3.78	2.35	2.43	Omega-6 fatty acid desaturase	LOC_Os07g23410	+
Os07g0537900	1.12	1.24	1.83	1.62	Ser/Thr kinase receptor domain	LOC_Os07g35340	
Os07g0664000	1.52	2.24	2.59	1.85	Putative short chain dehydrogenase/reductase	LOC_Os07g46870	+
Os08g0203400	1.66	4.69	2.54	3.59	Protein kinase/core domain containing protein	LOC_Os08g10310	
Os08g0501500	1.03	1.27	2.26	1.70	OsWAK receptor-like protein kinase	LOC_Os08g39210	+
Os09g0358000	1.77	3.07	2.71	2.51	Tyrosin kinase domain containing protein	LOC_Os09g19350	
Os09g0358100	1.27	2.05	1.79	1.05	Senescence-induced serine/threonine kinase	LOC_Os09g19360	
Os10g0195250	1.24	2.08	2.01	1.68	Conserved hypothetical protein	LOC_Os10g11889	
Os11g0262600	1.31	2.45	2.29	2.29	Conserved hypothetical protein	LOC_Os11g15624	
Os11g0474800	2.83	2.81	3.20	2.57	Putative stemar-13-ene synthase	LOC_Os11g28530	
Os12g0171801	1.12	2.77	2.75	1.64	Hypothetical protein	None	
Os12g0236100	1.17	2.03	1.70	1.60	Conserved hypothetical protein	LOC_Os12g13340	
Os12g0555200	2.22	3.97	2.77	3.31	Putative pathogenesis-related Bet v family protein	LOC_Os12g36850	+

a *Genes previously shown to be induced in rice root 6 days post-inoculation with M. oryzae (Marcel et al., 2010) are indicated with the sign +*.

Making use of the Rice Oligonucleotide Array Database (Cao et al., 2012), we identified experiments analyzing gene expression in rice during plant-microbe interactions. Prior to this analysis, RT-qPCR expression profiles observed in rice roots for the 14 genes used to validate our microarray data (Table 1) were compared to RT-qPCR expression profiles obtained in rice leaves and none displays differential expression when considering shoot compartment (data not shown). Then, the array database analysis was focused on rice roots and only one experiment analyzing gene expression in the root compartment was evidenced. This work considered the impact of the fungus phytopathogen *Magnaporthe oryzae* on rice transcriptome 6 days after infection (Marcel et al., 2010). The list of 34 potential markers of *Azospirillum*-rice cooperation, up-regulated in the four combinations, was compared to the list of genes differentially expressed after *M. oryzae* inoculation, evidencing that 10 of the potential markers (3 genes of unknown function) are also up-regulated in response to *Magnaporthe* infection while none of these markers are down-regulated after *Magnaporthe* infection (Table 2). Thus, the PR gene orthologous to PR10 of *Brachypodium* (Os12g0555200) discussed above, as well as a gene related to signal transduction (Os08g0501500) and a gene encoding a Cys-Rich domain containing protein (Os02g057980) are up-regulated in response to both *Azospirillum* and *Magnaporthe*.

To understand whether rice biotic stress responses could be specific to either host or non-host interactions, the analysis was focused on the comparison of host combinations and non-host combinations. According to Figure 1, the number of up-regulated genes appears to be lower when a strain is inoculated on its original host cultivar (Cig_4B and Nip_B510). In addition, fewer genes implicated in response to stress and plant defense are differentially expressed in host combinations (Figure 2), the most striking example being PR genes as highlighted in Figure 3. However, among all differentially expressed genes, only one down-regulated gene is common to both host combinations (Os07g0638600 encoding a peroxidase) and 55 genes (53 up-regulated, 2 down-regulated) are common to non-host combinations (Figure 1). Among the latter, two genes implicated in successive steps of ethylene synthesis (Os06g0524900, Os09g0451400), a NB-ARC domain containing gene (Os06g0524900) and a gene encoding an expansin (Os05g0276500) were identified. Several genes implicated in oxido-reductive processes (Os01g0327000, Os07g0164900, Os12g0260500) as well as a gene encoding a glycoside hydrolase (Os09g0395600) and a gene encoding an UDP-glucuronosyl transferase (Os01g0597800) are also induced. When comparing to the list of genes differentially expressed after *Magnaporthe* infection, 6 of the 53 up-regulated genes common to both *Azospirillum*-rice non-host combinations (Cig_B510 and Nip_4B) are also induced after *Magnaporthe* inoculation and none of these genes are repressed (Additional File 6: Table S6). However, these genes do not seem to be directly involved in biotic stress response of rice roots.

### Combination-specific expression profiles

Many differences are observed between the four cultivar/strain combinations when comparing expression profiles, particularly for genes involved in biotic stress responses (Figure 3). This includes genes potentially involved in hormone signaling and both ethylene and auxin signaling occur to be finely regulated during *Azospirillum*-rice cooperation.

Indeed, if only one of the genes related to ethylene signaling was classified as Cig_4B specific, 15 of these genes were classified as Cig_B510-specific, 11 were classified as Nip_4B-specific and 13 were classified as Nip_B510-specific (Table 3). All the Cig_4B- and Nip_4B-specific genes related to ethylene signaling are up-regulated, while two of the Cig_B510-specific genes and all the Nip_B510-specific genes related to ethylene signaling are down-regulated. When considering genes related to auxin signaling, one was classified as Cig_4B specific, seven were classified as Cig_B510-specific, 12 were classified as Nip_4B-specific and eight were classified as Nip_B510-specific. In addition, nine genes involved in abscisic acid signaling and seven genes involved in salicylic acid signaling were shown to be differentially expressed in only one of the four combinations, suggesting that a wide modification of hormone signals occurs in rice roots after *Azospirillum* inoculation. Auxin, ethylene, abscissic acid, and salicylic acid are known to be involved in plant immunity, and many genes related to biotic stress response display a combination-specific profile in the current study. For example, 76 genes similar to PR-genes were identified to be combination-specific. As previously mentioned, fewer genes are differentially expressed in host combinations than in non-host combinations. Indeed, three and 13 of these genes were identified for Cig_4B and Nip_B510, respectively, while 25 and 35 of these genes were identified for Nip_4B and Cig_B510, respectively. Taken all together, these results suggest that *Azospirillum* inoculation leads to important changes in rice root hormone signaling and plant defense, depending on the strain/cultivar combination.

**Table 3:** **List of combination-specific genes related to ethylene signaling**.

**Gene ID**	**Log2(FC)**				**Genome annotation**
	**Cig_4B**	**Cig_B510**	**Nip_4B**	**Nip_B510**	
Os02g0594300	1.29				Similar to enhancer of shoot regeneration ESR1
Os01g0797600		−1.77			AP2 domain-containing ethylene responsive protein
Os01g0536400		−1.66			Similar to 1-aminocyclopropane-1-carboxylate oxidase
Os04g0257500		1.10			Similar to ethylene-responsive transcription factor TSFR1
Os09g0451000		1.17			Similar to 1-aminocyclopropane-1-carboxylate oxidase
Os10g0523900		1.19			AP2 domain containing ethylene responsive protein
Os05g0437100		1.26			Ethylene-responsive transcription factor
Os01g0757200		1.30			Similar to gibberellin 2-beta-dioxygenase
Os03g0860600		1.35			Similar to 1-aminocyclopropane-1-carboxylate oxidase
Os02g0767300		1.36			Similar to flavonol synthase
Os09g0570800		1.41			Similar to 1-aminocyclopropane-1-carboxylate oxidase
Os01g0832600		1.66			Similar to leucoanthocyanidin dioxygenase
Os09g0248900		1.70			Similar to ethylene-responsive protein
Os02g0797100		1.89			AP2 domain containing ethylene responsive protein
Os05g0127500		1.95			Similar to leucoanthocyanidin dioxygenase
Os04g0182200		2.60			2OG-Fe(II) oxygenase domain containing protein
Os01g0230200			1.06		Ethylene-responsive protein
Os07g0169600			1.12		2OG-Fe(II) oxygenase domain containing protein
Os03g0100900			1.15		Ethylene-responsive element-binding protein
Os10g0536400			1.18		2OG-Fe(II) oxygenase domain containing protein
Os04g0407800			1.24		2OG-Fe(II) oxygenase domain containing protein
Os06g0162500			1.29		Similar to Naringenin 3-dioxygenase like protein
Os02g0202000			1.32		Similar to ethylene responsive protein
Os04g0548000			1.32		Ethylene-responsive element-binding protein
Os02g0654700			1.50		Ethylene-responsive transcription factor
Os03g0122300			1.53		Similar to Flavanone 3-hydroxylase-like protein
Os08g0366100			1.75		Endothelial differentiation-related factor
Os06g0177600				−2.07	2OG-Fe(II) oxygenase domain containing protein
Os11g0186900				−1.82	Similar to 1-aminocyclopropane-1-carboxylate oxidase
Os04g0522500				−1.58	2OG-Fe(II) oxygenase domain containing protein
Os05g0155200				−1.41	Similar to ethylene receptor
Os01g0935400				−1.41	2OG-Fe(II) oxygenase domain containing protein
Os02g0520000				−1.41	Ethylene responsive protein
Os04g0643500				−1.30	2OG-Fe(II) oxygenase domain containing protein
Os02g0276900				−1.28	Ethylene-responsive protein
Os04g0565900				−1.22	Ethylene-responsive protein
Os04g0493100				−1.21	Ethylene-responsive protein
Os04g0578000				−1.18	Similar to 1-aminocyclopropane-1-carboxylate synthase
Os03g0690500				−1.16	Similar to 1-aminocyclopropane-1-carboxylate oxidase
Os06g0573900				−1.10	Similar to 1-aminocyclopropane-1-carboxylate oxidase

Besides genes related to hormone signaling, the combination-specific response also includes nine genes related to hydroxycinnamic acids metabolism (Additional File 3: Table S3). This includes seven genes encoding cinnamyl alcohol dehydrogenase (Os01g0528800, Os03g0223200, Os09g0400200, Os11g0622800 which are down-regulated; Os04g0612700, Os09g0399800, Os09g0400400 which are up-regulated), one gene encoding a cinnamoyl-CoA reductase (Os01g0828100, downregulated) and one gene encoding a coumarate-CoA synthase (Os08g0245200, downregulated). In addition, four genes related to chalcones metabolism and five genes related to anthocyanin metabolism display a combination-specific profile. Such a result is of particular interest as some secondary metabolites stemming from these pathways were previously shown to be discriminant in the analysis of root methanolic extract composition, following *Azospirillum* and *Frankia* inoculations (Popovici et al., 2011; Chamam et al., 2013).

## Discussion

Unraveling the molecular basis of host-specific adaptations in PGPR-plant cooperation helps understanding frontiers between the perception of symbiotic, cooperative, and pathogenic microbes hosted by plant roots. Based on previous studies detecting metabolic and morphological changes of PGPR-inoculated plants at early stages (Cassán et al., 2009; Walker et al., 2011, 2012; Chamam et al., 2013), we analyzed the transcriptomic response of rice roots 7 days after inoculation. To our knowledge, this study constitutes the first investigation of wide transcriptomic response of rice roots to PGPR-inoculation, aiming at deciphering interaction specificity in plant-microbe cooperation.

Comparison of expression profiles obtained for Cigalon/*A. lipoferum* 4B (Cig_4B), Cigalon/*Azospirillum* sp. B510 (Cig_B510), Nipponbare/*A. lipoferum* 4B (Nip_4B) and Nipponbare/*Azospirillum* sp. B510 (Nip_B510) combinations evidences a fine-tuned transcriptomic response depending on both *Azospirillum* and rice genotypes. As revealed by the high percentage (83%) of genes up-regulated or down-regulated only in one of the four conditions, individual genotypic variations are the most important driving force of rice roots gene expression, in the tested conditions. Indeed, only 34 markers of the *Azospirillum*-rice cooperation, induced in the four combinations, were identified and further investigations should be undertaken to identify the impact of a larger range of *Azospirillum* strains on the regulation of these markers. Besides combination-specific traits, expression profiles showed strain-specific and cultivar-specific characteristics, highlighting potential differences in the strategies of interaction. Indeed, strain 4B causes few repressions while at least half of the genes regulated in response to strain B510 are repressed, regardless of the cultivar. In addition, only 59 genes display similar regulation in both combinations involving strain 4B whereas it represents 626 genes for those involving strain B510. Accordingly, strain-specific responses were observed when considering the respective impact of 4B and B510 on development and secondary metabolism of rice cultivars Cigalon and Nipponbare (Chamam et al., 2013). While 4B promotes shoot and root growth of both cultivars, B510 promotes development of cultivar Nipponbare exclusively. However, B510 was shown to be the only strain inducing a systemic response, as revealed by variation of secondary metabolite profiles of both shoots and roots. These strain-specific responses could be due to differences in strain lifestyle as 4B colonizes only the surface of rice roots while B510 has the ability to colonize the cortex layers in rice (Thomas-Bauzon et al., 1982; Elbeltagy et al., 2001; Chamam et al., 2013). In addition to their impact on plant growth, endophytic PGPR induce stress and defense responses and the inoculation of B510 was shown to enhance resistance against rice blast disease and rice blight disease (Miché et al., 2006; Rosenblueth and Martínez-Romero, 2006; Yasuda et al., 2009). However, whether these changes were the result of major gene induction or repression was not addressed. Recently, a study on differential gene expression of rice roots inoculated with the endophyte *Herbaspirillum seropedicae* evidenced a decrease in expression of defense related protein PBZ1 and thionins, suggesting that bacteria modulate plant defense to allow the establishment of an efficient cooperation (Brusamarello-Santos et al., 2011). Indeed, colonization of root tissues by bacteria depends on the balance between the plant's ability to induce efficient defenses in response to the intrusive microbe and the microorganisms' ability to bypass plant immunity (Pieterse et al., 2009).

Several genes associated to plant defense mechanisms are still regulated 7 days post-inoculation. Particularly, a gene orthologous to the PR10 gene of *Brachypodium* is induced in all combinations. Interestingly, this gene was previously shown to be up-regulated in rice roots 6 days after *Magnaporthe* infection (Marcel et al., 2010). PR genes are known to be induced during PAMPs-triggered immunity (PTI), the first step of plant defense that involves Pattern Recognition Receptors (PRRs) (Chisholm et al., 2006; Pieterse et al., 2009). PRRs recognize universal microbial determinants such as flagellin, chitin, glycoproteins, and lipoproteins designed as PAMPs/MAMPs for Pathogens/Microbes–Associated Molecular Patterns (Schwessinger and Zipfel, 2008). Even if symbiotic and pathogenic interactions exhibit a number of unique characteristics, it was suggested that similar response mechanisms were adapted to cope with different biotic and abiotic stresses (Baron and Zambryski, 1995). Surprisingly, down-regulated mechanisms seem to be more conserved than up-regulated mechanisms between pathogenic and symbiotic interactions, genes involved in plant defense and stress response being a major part of these repressions (Damiani et al., 2012). In this context, endophytic colonization being more intrusive than surface colonization, plant response to endophyte PGPR could be more similar to pathogenic response than plant response to surface colonizers. This hypothesis is supported by the fact that 30 genes common to Cig_B510 and Nip_B510 are also down-regulated during *Magnaporthe* infection while none of the genes common to Cig_4B and Nip_4B are down-regulated (data not shown).

Most of the genes containing a NB-ARC domain are differentially expressed on a strain/cultivar dependent manner, the most striking effect being observed for the non-host combinations. Most of NB-ARC genes regulated during interaction with strain B510 are down-regulated and repression occurs for a higher number of NB-ARC genes in the Nip_B510 host interaction. NB-ARC domain is generally associated to R genes that are involved in Effector-Triggered Immunity (ETI) (Chisholm et al., 2006; Pieterse et al., 2009). Differences observed between host and non-host combinations suggests that the way a strain is perceived by rice roots could have been subjected to long-lasting co-adaptation events between a strain and its original host cultivar, a hypothesis that has already been proposed based on secondary metabolites profiling of rice inoculated with strain 4B and B510, and on transcriptomic response of strain 4B colonizing rice (Chamam et al., 2013; Drogue et al., 2014). The involvement of plant defense systems in PGPR-plant cooperations was mainly considered in the context of biocontrol agents, their perception leading to the induction of long-lasting and broad-spectrum systemic resistance (Van Loon et al., 1998; Van Wees et al., 2008; Pieterse et al., 2009). Induced systemic resistance (ISR) is associated with priming effect for enhanced defense inducing a few reprogramming of plant transcriptome (Verhagen et al., 2004; Wang et al., 2005; Van Wees et al., 2008). In the case of phytostimulating PGPR, it was reported that members of genus *Azospirillum* and *Burkholderia* induce defense response at a lower extent than pathogens (Bashan, 1998; Bordiec et al., 2011). Moreover, induction of plant defense mechanisms was shown to control the establishment of compatible and incompatible interactions between plants and endophytic PGPR (Miché et al., 2006; Rosenblueth and Martínez-Romero, 2006; Reinhold-Hurek and Hurek, 2011). Thus, discrepancies between endophyte and surface colonizer effects on plant defense system should be further investigated by studying the impact of other *Azospirillum* strains that display endophytic properties, such as *A. brasilense* Sp245. A recent study analyzed the impact of *A. brasilense* Sp245 inoculation on *A. thaliana* gene expression and evidenced that root transcriptome undergoes significant changes on genes related to hormone signaling and plant defense (Spaepen et al., 2014) Taking into account that plant immunity involves dynamic mechanisms that lead to temporal changes in the expression of defense related genes remains an important issue to measure the sustainability of PGPR-plant cooperations.

Modulating plant hormone balance is an important trait of phytostimulating PGPR (Richardson et al., 2009; Bashan and de-Bashan, 2010; Vacheron et al., 2013). Especially, several members of the genus *Azospirillum* are able to produce auxin, cytokinin, and gibberellin (Richardson et al., 2009; Bashan and de-Bashan, 2010). In the case of strains 4B and B510, genes related to indole-3-acetic acid (IAA) biosynthesis pathway, *ipdC*/*ppdC*, are absent from their genomes and 1-aminocyclopropane-1 carboxylate (ACC) deamination could be a relevant mechanism for hormone modulation and plant-growth promotion (Blaha et al., 2006; Prigent-Combaret et al., 2008; Kaneko et al., 2010; Wisniewski-Dyé et al., 2011). This property is encoded by *acdS* found in both pathogenic and non-pathogenic bacteria (Blaha et al., 2006). ACC is a precursor of ethylene, a gaseous hormone that represses root-growth and induces systemic resistance against pathogens (Bleecker and Kende, 2000; Pieterse et al., 2009; Galland et al., 2012). It was proposed that bacterial deamination of ACC could lead to a decrease of ethylene levels in plant roots and consequently an increase in root development (Glick, 2005; Galland et al., 2012). Interestingly, the impacts of strain 4B and strain B510 on rice root morphological traits differ. Indeed, strain 4B improves total root length, principal root length and the number of roots per plant while strain B510 seems to improve mostly principal root length (Chamam et al., 2013). Then, differential impact of strain/cultivar combinations on rice root architecture could be linked to the combination-specific regulation of many genes related to ethylene biosynthesis.

Many plant hormones are involved in both plant growth and plant immunity, two physiological traits regulated by a network of interconnected signaling pathways (Pieterse et al., 2009). As such, auxin contributes to both plant development and disease resistance in a pathway interconnected with salicylic acid signaling (Wang et al., 2007). Cross-communication between plant immunity and plant development may contribute to quick adaptation in a cost-efficient manner according to numerous trade-offs reported between growth rate and disease resistance (Walters and Heil, 2007; Pieterse et al., 2009). Thus, the strain-specific effect of *Azospirillum* on the regulation of hormone-related genes must be taken into account to appraise the cost-benefit balance of each strain/cultivar cooperation. While these two strains display similar characteristics for hormonal production (Wisniewski-Dyé et al., 2012), regulation of rice genes related to hormone signaling, notably auxin signaling, is strikingly different when considering each strain (see above). These results highlight the complexity of hormone signaling networks involved in *Azospirillum*-rice cooperation.

This study aimed at identifying genetic determinants regulated in rice roots upon *Azospirillum* inoculation, considering possible favored interaction between a strain and its original host cultivar. Thus, two rice cultivars were inoculated with two *Azospirillum* strains, resulting in four strain/cultivar combinations. The wide set of genes differentially expressed only in one of the four combinations suggest that individual genotypic variations could be the most important driving force of rice root gene expression upon *Azospirillum* inoculation. Strain-dependent transcriptional changes observed for genes related to auxin and ethylene signaling highlight the complexity of hormone signaling networks in the *Azospirillum*-rice cooperation. In this context, unraveling cross-connected hormone networks involved in both growth promotion and plant defense response appears to be an important issue to understand mechanisms involved in beneficial interactions between PGPR and plants.

## Author contributions

Florence Wisniewski-Dyé and Claire Prigent-Combaret conceived and designed the experiments with the help of Nathalie Picault. Amel Chamam and Hervé Sanguin performed the experiments and Benoît Drogue contributed to the preparation of rice RNA samples. Benoît Drogue and Hervé Sanguin performed the analysis of microarray data, with the help of Michael Mozar for the analysis of raw data. Christel Llauro, Nathalie Picault, and Olivier Panaud performed the qRT-PCR and the part on LTR-retrotransposons. Benoît Drogue, Hervé Sanguin, Claire Prigent-Combaret, and Florence Wisniewski-Dyé wrote the manuscript; all authors contributed to the discussion and approved the final manuscript.

### Conflict of interest statement

The authors declare that the research was conducted in the absence of any commercial or financial relationships that could be construed as a potential conflict of interest.
